# Sirt1 Regulates Insulin Secretion by Repressing UCP2 in Pancreatic β Cells

**DOI:** 10.1371/journal.pbio.0040031

**Published:** 2005-12-27

**Authors:** Laura Bordone, Maria Carla Motta, Frederic Picard, Ashley Robinson, Ulupi S Jhala, Javier Apfeld, Thomas McDonagh, Madeleine Lemieux, Michael McBurney, Akos Szilvasi, Erin J Easlon, Su-Ju Lin, Leonard Guarente

**Affiliations:** 1Department of Biology, Massachusetts Institute of Technology, Cambridge, Massachusetts, United States of America; **2**Laval Hospital Research Center, Québec City, Québec, Canada; **3**The Whittier Institute for Diabetes, University of California San Diego, La Jolla, California, United States of America; **4**Elixir Pharmaceuticals, Cambridge, Massachusetts, United States of America; **5**Department of Medicine and Department of Biochemistry, Microbiology, and Immunology, University of Ottawa, and the Ottawa Regional Cancer Centre, Ottawa, Ontario, Canada; **6**Novartis Institutes for BioMedical Research, Cambridge, Massachusetts, United States of America; **7**Center for Genetics and Development, and Section of Microbiology, University of California Davis, Davis, California, United States of America; The Salk InstituteUnited States of America

## Abstract

Sir2 and insulin/IGF-1 are the major pathways that impinge upon aging in lower organisms. In *Caenorhabditis elegans* a possible genetic link between Sir2 and the insulin/IGF-1 pathway has been reported. Here we investigate such a link in mammals. We show that Sirt1 positively regulates insulin secretion in pancreatic β cells. Sirt1 represses the uncoupling protein (UCP) gene UCP2 by binding directly to the UCP2 promoter. In β cell lines in which Sirt1 is reduced by SiRNA, UCP2 levels are elevated and insulin secretion is blunted. The up-regulation of UCP2 is associated with a failure of cells to increase ATP levels after glucose stimulation. Knockdown of UCP2 restores the ability to secrete insulin in cells with reduced Sirt1, showing that UCP2 causes the defect in glucose-stimulated insulin secretion. Food deprivation induces UCP2 in mouse pancreas, which may occur via a reduction in NAD (a derivative of niacin) levels in the pancreas and down-regulation of Sirt1. Sirt1 knockout mice display constitutively high UCP2 expression. Our findings show that Sirt1 regulates UCP2 in β cells to affect insulin secretion.

## Abstract 2

Sirt1 is shown to regulate the expression of the metabolic decoupling gene UCP2 in pancreatic β cells, highlighting a possible role for Sirt1 in coordinating insulin release in response to changing dietary conditions.

## Introduction

Glucose homeostasis is maintained, in part, by pancreatic β cells, which secrete insulin in a highly regulated sequence of dependent events [1]. β cells metabolize glucose, resulting in an increase in the ATP/ADP ratio, the closing of the ATP-dependent K^+^ channel, the activation of the voltage-gated Ca^+^ channel and Ca^+^ influx, and the fusion of secretory vesicles to the plasma membrane to release insulin. Insulin is part of an organismal physiological axis in which it stimulates glucose uptake in metabolic tissues, such as muscle, and stores energy in the form of fat in white adipose tissue (WAT). Short-term food limitation (i.e., overnight [O/N] fasting) will therefore elicit the mobilization of glycogen stores and then fat from WAT for metabolism, and the lower level of blood glucose during fasting will result in low levels of insulin production by β cells.

Long-term calorie restriction (CR) has been known for 70 years to extend the life span of mammals dramatically [2], and it can also work in a variety of organisms, including yeast, flies, and rodents [3–5], although the mechanism of this effect has remained obscure. In mammals a characteristic set of physiological changes takes place during long-term CR, which overlaps the rapid physiological adaptations to short-term food limitation. One such change is the use of dietary fat or fat mobilized from WAT for energy [4]. Another is a large reduction in blood insulin levels accompanied by an increase in insulin sensitivity, i.e., the ability of insulin to promote glucose utilization [4]. In addition, gluconeogenesis is activated in the liver. These changes keep glucose available for the brain, and are closely associated with the longevity elicited by CR. The paucity of fat in WAT appears to be sufficient per se to promote a degree of longevity, since mice engineered for leanness—for example, a WAT-specific knockout (KO) of the insulin receptor—live longer [6,7].

Findings in model organisms suggest a mechanism for the longevity engendered by CR that implicates the silent mating type information regulation 2 gene *(Sir2).* This gene regulates the life span in yeast [8] and *Caenorhabditis elegans* [9] as a longevity determinant. In yeast, CR works by up-regulating the activity of Sir2 [10,11], a NAD-dependent deacetylase [12–14] (NAD is a derivative of niacin), by increasing respiration, and by increasing the NAD/NADH ratio [15] (NADH is the reduced form of NAD). CR is also reported to activate the NAD salvage pathway, which would deplete a Sir2 inhibitor, nicotinamide [3,10]. The *Drosophila melanogaster Sir2* gene was also shown to mediate life extension in response to dietary restriction [16,17].

Since Sir2 appears to mediate the effects of CR on life span in simple model organisms, it seemed possible that Sir2 proteins also regulate the effects of food limitation and CR in mammals. The homolog of the yeast silencing information regulator2 (Sirt1) has also been implicated in several aspects of food limitation and CR in mammals. In WAT, Sirt1 represses the key regulatory protein peroxisome proliferator-activated receptor gamma (PPARγ), resulting in fat mobilization in response to food limitation [18]. In addition, Sirt1 regulates the FOXO (forkhead Box O) set of forkhead transcription factors [19,20], providing another link to metabolism and diet. Also, gluconeogenesis in the liver is regulated by Sirt1 [19], which works in concert with the transcriptional co-activator, peroxisome proliferator-activated receptor coactivator, PGC-1α [21]. Finally, Sirt1 may play a role in the observed stress resistance of CR animals, since it down-regulates several pro-apoptotic factors, such as p53, FOXO, and Bax [19,20,22–25].

In addition to the classical paradigm for insulin regulation by glucose outlined above, reports suggest a role of an uncoupling protein (UCP) in insulin secretion. UCPs belong to a family of mitochondrial inner membrane proteins. They function to uncouple oxygen consumption during respiration from the production of ATP by allowing proton leakage down an electrochemical gradient from the cytoplasm to the mitochondria [26–29]. Several mammalian UCP homologues have been identified and characterized [28]. UCP2 has been shown to promote proton leakage across the mitochondrial membrane [30–33], and has been proposed to play a role in lipid metabolism, insulin resistance, glucose utilization, regulation of reactive oxygen species, and macrophage-mediated immunity [28,34,35]. A role for UCP2 in insulin secretion has been demonstrated, since UCP2 KO mice show higher ATP levels in islets and increased insulin secretion upon glucose stimulation [36]. Conversely, UCP2 overexpression in cultured β cell lines reduces ATP levels and glucose-stimulated insulin secretion [37,38].

In this study, we show that Sirt1 functions as a positive regulator of insulin secretion in response to glucose by directly repressing the UCP2 gene. Our findings suggest that UCP2 is thus used in β cells to modulate the insulin response pathway as a function of diet. The fact that Sirt1 is a positive regulator of insulin may seem surprising, in light of the fact that *sir-2.1* in *C. elegans* appears to be a negative regulator of the insulin-like signaling pathway [9]. We discuss how this difference makes sense in light of the physiological adaptations that take place in mammals in response to dietary changes.

## Results

### Sirt1 Is Preferentially Expressed in Pancreatic β Cells

**Figure 1:**
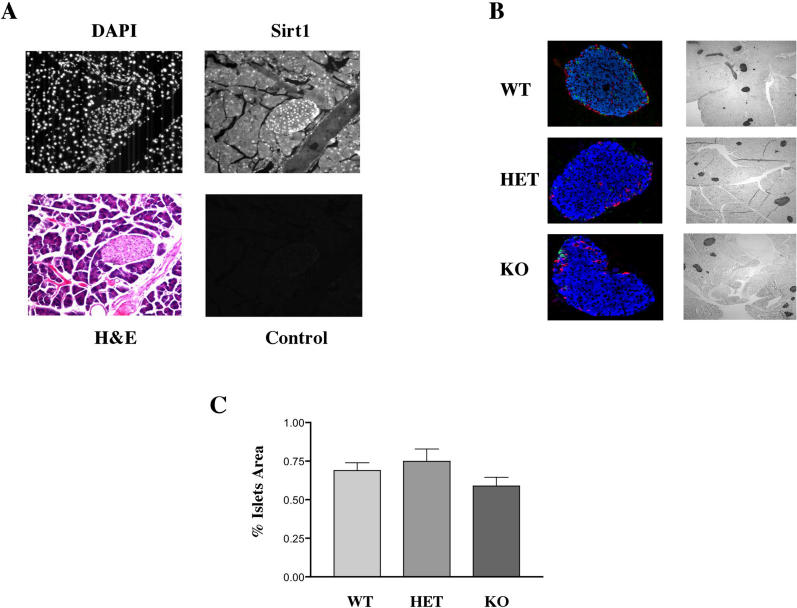
Sirt1 Is Localized in the Islets of Langerhans The pancreas of wild-type mice was sectioned and stained as described. (A) Nuclear staining using DAPI (top left). Immunofluorescence using Sirt1 antibody (top right); hematoxylin and eosin staining of the same section of pancreas (bottom left); immunofluorescence control using a rabbit secondary antibody (bottom right). (B) Pancreases of wild-type (WT), Sirt1+/− heterozygotes (HET), or Sirt1−/− homozygous KO mice were stained with antibodies against insulin (blue), glucagon (red), or somatostatin (green) (shown in left column). Representative islets of mice of all three genotypes are shown. Pancreases were also silver-stained for morphometry (right column). Islets appear as dark figures and their area was determined by scanning, using Image-Pro 4.1 Plus software. (C) The areas are shown as percentage of area of the entire pancreas.

To determine whether Sirt1 could potentially play a role in β cells, immunofluorescence of whole murine pancreas with anti-Sirt1 antibody was carried out, and 20 islets were examined by fluorescence microscopy. A representative islet is shown in Figure 1A (see lower left panel for hematoxylin-eosin staining). All pancreatic sections examined showed intense staining concentrated in islets using Sirt1 antibodies (Figure 1A, top right) but not with secondary antibody alone (Figure 1A, lower right), and a lower level of expression in the surrounding exocrine cells. DAPI bright spots (Figure 1A, top left) mark nuclei and their corresponding cells in the islets and surrounding tissue. This enrichment of Sirt1 in the islets and not exocrine cells is noteworthy, in light of the ubiquitous expression of this sirtuin in most somatic and germ tissues [39,40].

### Sirt1 KO Mice Have Low Levels of Blood Insulin

Because Sirt1 is highly expressed in β cells, we investigated whether this sirtuin plays a functional role in insulin production. First, we determined whether Sirt1 KO mice [39,40] showed any defects in the pancreatic β cells or the islets. Pancreases from wild-type, Sirt1+/− heterozygotes, or Sirt1−/− KO mice (4−5 each) were sectioned and stained with antibodies against insulin (blue), glucagons (red), and somatostatin (green). These stains mark islets for β cells, α cells, and δ cells, respectively. As shown in a typical section (Figure 1B), no differences were observed in the staining pattern of these three markers between wild-type and mutant islets. We measured pancreatic islet areas using Image-Pro 4.1 Plus software, and expressed the islet areas as a percentage of the total pancreatic area (Figure 1C). There were no significant differences in islet areas comparing wild-type, heterozygous, and KO mice. The absolute islet size was also not appreciably different in the mutant mice.

**Figure 2:**
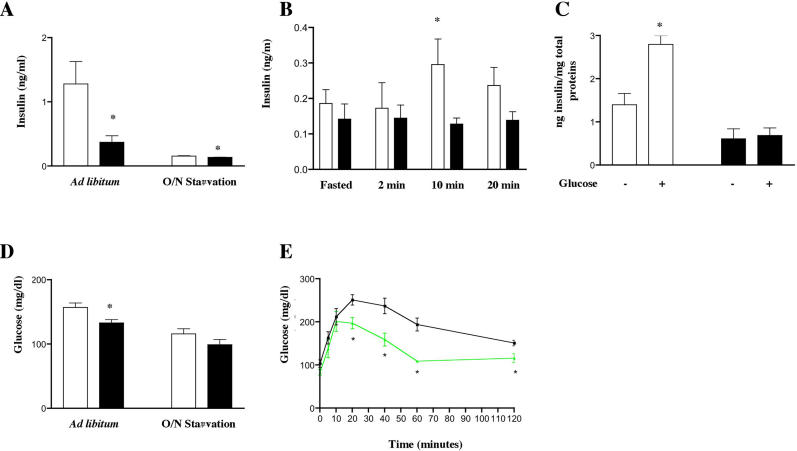
Sirt1 KO Mice Have a Lower Level of Insulin (A) Plasma insulin levels in wild-type (open bars) or Sirt1 KO mice (black bars) *ad libitum* or after O/N starvation (*n* = 12 wild-type, 11 KO, *p < 0.03 in *ad libitum* and O/N starvation mice, ANOVA). (B) Plasma insulin levels in Sirt1 KO mice (black bar) compared with wild-type mice (open bars) 2, 10, or 20 min after injection with glucose (*n* = 4 or 5, *p < 0.05 compared with wild-type, ANOVA). (C) Insulin secretion in islets isolated from wild-type (open bars) or Sirt1 KO mice (black bars) after induction by 20 mM glucose for 1 h (*n* = 4, *p < 0.005 in wild-type, ANOVA). (D) Glucose levels in wild-type (open bars) and Sirt1 KO (black bars) mice (*n* = 12 wild-type, 11 KO, *p < 0.03 *ad libitum,* ANOVA). (E) Glucose tolerance tests in wild-type (black) and Sirt1 KO (green) mice (*n* = 6, *p < 0.05 at 20, 40, 60, and 120 min).

Next, we determined whether insulin production was altered in Sirt1 KO mice. Blood insulin was measured in males 2–4 mo old fed *ad libitum* or after 18 h of starvation (Figure 2A). Sirt1 KO mice (black bars) had much lower blood levels of insulin compared with littermate control animals (open bars) when the samples were collected under *ad libitum* conditions. This difference was also observed when the animals were starved O/N, in which case insulin levels were very low in both wild-type and KO mice. To assess more precisely the ability of animals to produce insulin, mice were given an injection of glucose after O/N starvation and insulin was measured after 2, 10, and 20 min. Whereas insulin induction was clearly evident in the wild-type mice at 10 min (open bars), induction was not observed in Sirt1 KO animals (black bars in Figure 2B, *n* = 4 or 5 for each measurement). A similar trend was noted at 20 min.

To further investigate the defect in insulin production, islets from four wild-type or four Sirt1 KO animals were isolated and incubated in vitro with or without glucose for 1 h, and insulin secretion was determined. As shown in Figure 2C, the basal level of insulin secreted into the media from islets of Sirt1 KO mice (black bars) was significantly lower than wild-type controls (open bars). Moreover, the islets isolated from Sirt1 KO mice were not induced to secrete insulin by glucose, while control islets were inducible, as expected.

Levels of blood glucose were then determined and, surprisingly, were lower in Sirt1 KO mice (Figure 2D). Further, these mice appeared to be hypermetabolic, since they ate more food per body weight than the wild-type (unpublished data). These findings suggested that the KO mice had a better ability to use the lower levels of insulin for glucose uptake, i.e., were more insulin sensitive. To address this possibility, we performed glucose tolerance tests in both wild-type and KO mice by injecting glucose intraperitoneally and measuring the kinetics of glucose clearance from the blood. The KO mice cleared the glucose significantly faster than the wild-type (Figure 2E). This increased glucose tolerance explains why the KO mice maintained lower levels of blood glucose, even with reduced levels of insulin. The surprising effect of knocking out Sirt1 on glucose tolerance likely derives from tissues other than the pancreas and is currently being investigated in greater detail. In summary, we do not know whether the reduction in insulin in Sirt1 KO mice is due to a β cell defect or is an indirect consequence of increased glucose tolerance in these animals.

### Sirt1 Drives Glucose-Induced Insulin Secretion in Cultured Cells

**Figure 3:**
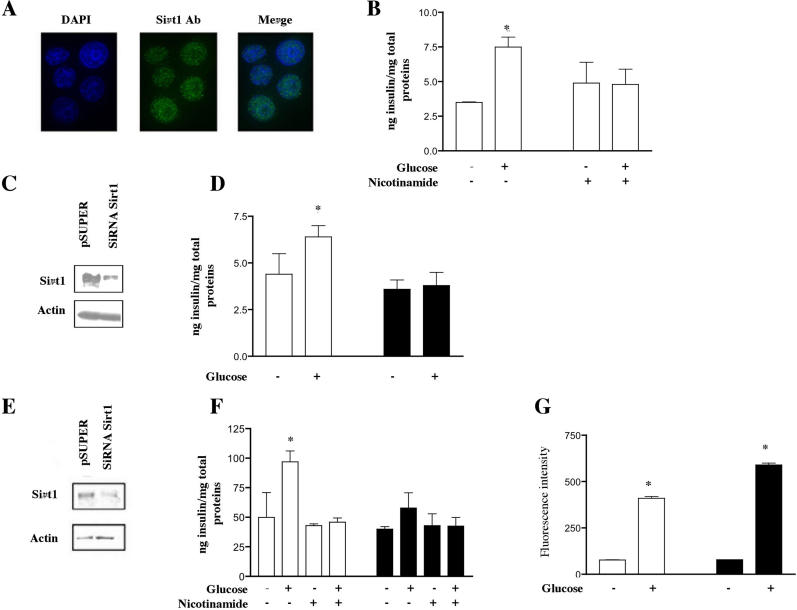
Sirt1 Is a Positive Regulator of Insulin Secretion in INS-1 and MIN6 Cells (A) Immunofluorescence in INS-1 cells using Sirt1 antibody (green) and DAPI staining (blue). Nuclear localization of Sirt1 is evident. (B) Induction of insulin secretion in INS-1 cells with 16.7 mM glucose (+) compared with 4 mM glucose control (−). The left side shows no nicotinamide and the right side shows treatment with 10 mM nicotinamide for 48 h prior to induction (*n* = 3 experiments done in triplicate, *p < 0.05 in the no nicotinamide experiment, ANOVA). (C) Western blot of Sirt1 in INS-1 cells with knockdown levels of the protein (SiRNA Sirt1) compared with control cells (pSUPER). (D) INS-1 cells infected with the pSUPERretro SiRNA-GFP control (open bars) or pSUPER retro SiRNA-Sirt1 knockdown cells (black bars) were induced for insulin secretion as in (B) (*n* = 3 experiments done in triplicate, *p < 0.008 in the control experiment, ANOVA). (E) Western blot of Sirt1 in MIN6 cells with knockdown levels of the protein (SiRNA Sirt1) compared with control cells (pSUPER). (F) Glucose induction (20 mM versus 4 mM) of insulin secretion in MIN6 cells with the pSUPER control vector (open bars) or the SiRNA Sirt1 vector (black bars) in the absence or presence of nicotinamide (*n* = 3 experiments done in triplicate*p < 0.05 in the control without nicotinamide, ANOVA). (G) Glucose uptake in INS-1 cells stably transfected with control or SiRNA Sirt1 vectors. 2-NBDG fluorescence was determined by flow cytometry 10 min after addition and expressed as arbitrary units (*n* = 2, *p < 0.0005 compared with no glucose).

To address whether Sirt1 played a positive role in insulin production specifically in β-cells, the pancreatic β-cell lines, INS-1 from rat, and MIN6 from mouse were employed. INS-1 cells were grown in culture, and nuclear expression of Sirt1 was evident by immunofluorescence (Figure 3) and Western blot (Figure 3C and 3E). INS-1 or MIN6 cells were treated with 10 mM nicotinamide, an inhibitor of Sirt1 [41], for 48 h, and the levels of insulin secreted into the media were measured without and with induction by glucose (Figure 3B and 3F, respectively). Whereas control cells showed robust induction of insulin secretion by glucose, nicotinamide treatment blocked this induction in both cell lines.

To test if the effects of nicotinamide were due specifically to inhibition of Sirt1, levels of this sirtuin were lowered by RNA interference. INS-1 and MIN6 cells were infected with a retrovirus carrying a Sirt1-SiRNA construct [18] or a control vector (pSUPER-SiRNA GFP), and stable cell lines were created as puromycin-resistant infected pools. These cells displayed Sirt1 protein levels that were knocked down compared with drug-resistant control pools generated from the vector (Figure 3C [INS-1] and 3E [MIN]). When these stable Sirt1 knockdown cells (Figure 3D, 3F, and 3G) were treated with glucose, induction of insulin secretion was eliminated, whereas contemporaneous assays of vector control cells (open bars) showed normal induction (Figure 3D and 3F).

We then tested if the defect in glucose-induced insulin secretion was due to a different glucose uptake between control and knockdown cells by measuring transport of fluorescence analog 2-NBDG. Glucose uptake [42,43] was not reduced and was perhaps slightly elevated in this assay in the knockdown cells (Figure 3G). Further, no decrease in the glucose transporter Glut 2 was observed (unpublished data). Finally, cell growth measurements revealed no difference in the growth rate of INS-1 knockdown cells compared with controls (Figure S1). These results show that knockdown of Sirt1 suppresses glucose-stimulated insulin secretion in two different β cell lines.

### ATP/ADP Ratio Is Lower in Sirt1 Knockdown Cells

**Figure 4:**
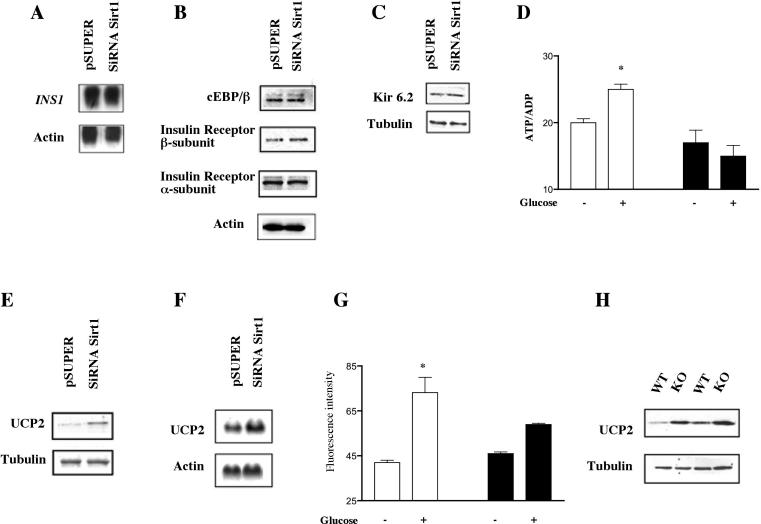
UCP2 is Up-Regulated in Sirt1 Knockdown Cells and in Sirt1 KO Mice (A) Northern blot for the insulin gene INS-1 in cells with a control vector (pSUPER) or a SiRNA-Sirt1 vector (SiRNA Sirt1). (B and C) Western blot analyses of targets involved in insulin synthesis and secretion using specific antibodies: cEBP/β, insulin receptor α and β, and kir6.2, one of the K^+^ channel receptor subunits. (D) Measurement of ATP/ADP levels in INS-1 control cells (open bars) or Sirt1 knockdown cells (black bars) treated with 16.7 mM glucose (+) or 4 mM glucose (−) (*n* = 3 experiments done in triplicate, *p < 0.005 in the pSUPER experiment; ANOVA). (E) Western blot analysis for UCP2 in INS-1 control cells (pSUPER) or knockdown cells (SiRNA Sirt1). (F) Northern blot analysis for UCP2 in INS-1 control cells (pSUPER) or knockdown cells (SiRNA Sirt1). (G) NADH levels in INS-1 cells after glucose addition as determined by autofluorescence [46] and expressed as arbitrary units. Cells stably transfected with control or Sirt1 SiRNA vectors were used (*n* = 2, *p < 0.05 compared with no glucose). (H) UCP2 protein levels in isolated pancreatic islets of two wild-type or two Sirt1 KO mice. Tubulin or actin was used as loading control in all Western and Northern blots.

We next sought to investigate the mechanism by which Sirt1 regulates insulin secretion in β cells. First, we assayed the RNA level of the *INS1* gene, encoding insulin, by Northern blot and found no difference in control versus Sirt1 knockdown β cells (Figure 4A). Similarly, we found no difference by Western blot in expression level of c/EBPβ, a transcription factor that regulates *INS1* (Figure 4B). Because the insulin receptor is part of an autocrine induction loop for insulin [44], we also determined by Western blot the levels of this receptor (α and β subunits) and observed no difference between control and knockdown cells (Figure 4B). Next, we investigated whether Sirt1 affected the levels of the K^+^ channel by Western blot, and again found no effect (Figure 4C). While this analysis suggests that Sirt1 does not function by altering levels of these factors, it does not rule out the possibility that Sirt1 regulates their activity.

Finally, because of the central role of ATP in insulin secretion, we surmised that Sirt1 could play a role in the energetics of glucose utilization in β cells. We thus measured the ATP/ADP ratio in control and Sirt1 knockdown INS-1 cells after glucose induction. Control cells (Figure 4D, open bars) responded to glucose by increasing the ratio of ATP to ADP, as expected (Figure 4D). In contrast, in cells with the knockdown levels of Sirt1 (Figure 4D, black bars), the ATP/ADP ratio did not increase upon glucose stimulation. These findings show that knocking down Sirt1 results in a defect in ATP production in response to glucose. Basal ATP levels are not significantly altered in knockdown cells, consistent with the fact that they grow as well as control cells.

### UCP2 Levels Are Increased in Sirt1 KO Mice and Knockdown Cells

One possibility for the failure of the Sirt1 knockdown cells to make ATP is that respiration is more uncoupled than in control cells, which would square well with the known link between the UCP2 and insulin production in β cells (36). Thus, we determined by Western blot the levels of UCP2 in control and Sirt1 knockdown cells. Strikingly, there was a significant increase in the UCP2 protein level in the knockdown cells (Figure 4E). This increase in protein level was mirrored by an increase in the mRNA in the same cells (Figure 4F), indicating that Sirt1 regulates UCP2 transcription. It was previously shown that UCP2 expression reduced NADH levels [45]. We therefore determined NADH levels by autofluorescence [46] and found a significantly lower level of NADH in the knockdown cells (Figure 4G).

Western blot for UCP2 in Sirt1 KO mice showed a similar effect. We observed an increase in UCP2 protein in the whole pancreas (unpublished data), which is a measure of the islets, since UCP2 is expressed in only the endocrine cells of the pancreas [38]. Moreover, UCP2 protein was also up-regulated in isolated islets of Sirt1 KO mice (Figure 4H). In summary, our expression studies suggest that Sirt1 is a repressor of UCP2 transcription in β cells, and by repressing this UCP, this sirtuin may allow cells to secrete insulin in response to glucose.

### Sirt1 Binds to the UCP2 Promoter

**Figure 5:**
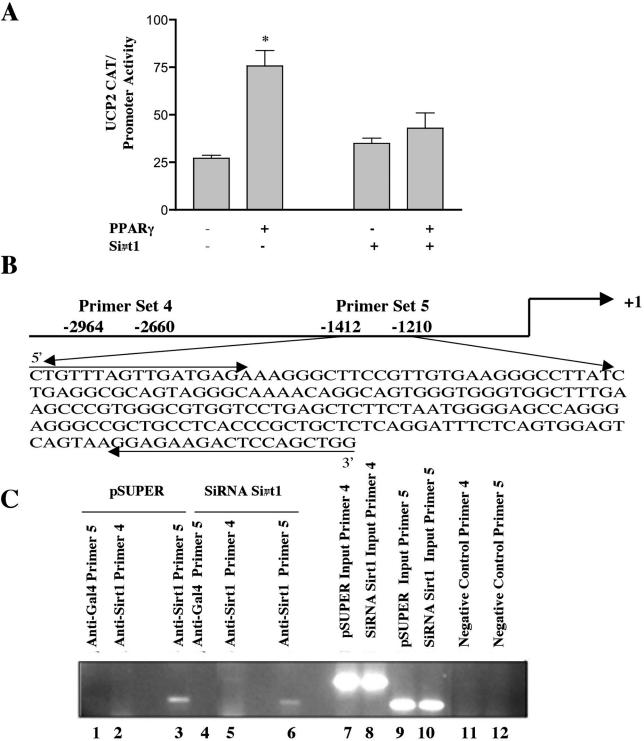
Sirt1 Binds at the UCP2 Promoter and Represses the Gene (A) In vitro CAT assay. 293T cells were transfected with a CAT reporter driven by the UCP2 promoter. Cells were also co-transfected with Sirt1 or not and with PPARγ or not, as indicated. CAT activity was determined (*n* = 3 experiments done in triplicate, *p < 0.05 in the no Sirt1 transfection experiment, ANOVA). (B) Schematic representation of the primer sets (arrows) in the UCP2 promoter (shown schematically and with excerpted DNA sequence). (C) Chromatin-immunoprecipitation (IP) was carried out on INS-1 control cells (lanes 1–3) or Sirt1 knockdown cells (columns 4–6) using Sirt1 antibody or a Gal4 control antibody, as indicated. PCR was carried out with the indicated primers. INPUT (columns 7–10) refers to PCR carried out on samples prepared prior to immunoprecipitation. Negative controls for the PCR (minus DNA) are also indicated (columns 11 and 12).

To study further the repression of UCP2 by Sirt1, we carried out reporter assays in 293T cells transfected with a chloramphenicol acetyl-transferase (CAT) gene, whose expression is driven by the UCP2 promoter. Cells were also transfected with a control vector or with an expression vector for PPARγ, which is known to bind to and activate the UCP2 promoter [47]. In a control experiment, PPARγ activated the reporter in this assay (Figure 5A, left bars). In a parallel experiment, cells were also co-transfected with a Sirt1 expression vector. Sirt1 clearly repressed the activation of the UCP2 promoter (Figure 5A, right bars). Repression of this reporter by endogenous or expressed Sirt1 was alleviated by nicotinamide, a known inhibitor of Sirt1 (unpublished data).

To determine whether repression of UCP2 was due to the direct binding of Sirt1 at the promoter, INS-1 cells were subjected to chromatin-immunoprecipitation, using Sirt1 or control antibodies. Primers specifically designed to span a known regulatory region of the UCP2 promoter (43) (Figure 5B, primer set 5) were used to probe by PCR the DNA in the immunoprecipitate. Sirt1 bound to this region of the UCP2 promoter in the control cells (Figure 5C, column 3) and to a significantly lesser extent in the Sirt1 knockdown cells (Figure 5C, column 6). Control primers designed in a different region of UCP2 upstream DNA (Figure 5B, primer set 4) showed no amplification (Figure 5C, columns 2 and 5). An anti-gal4 antibody control using primer set 5 also showed no binding (Figure 5C, columns 1 and 4). These findings indicate that Sirt1 represses UCP2 transcription by binding directly at the UCP2 promoter.

### Reduction of UCP2 Restores Insulin Secretion in Sirt1 Knockdown β cells

**Figure 6:**
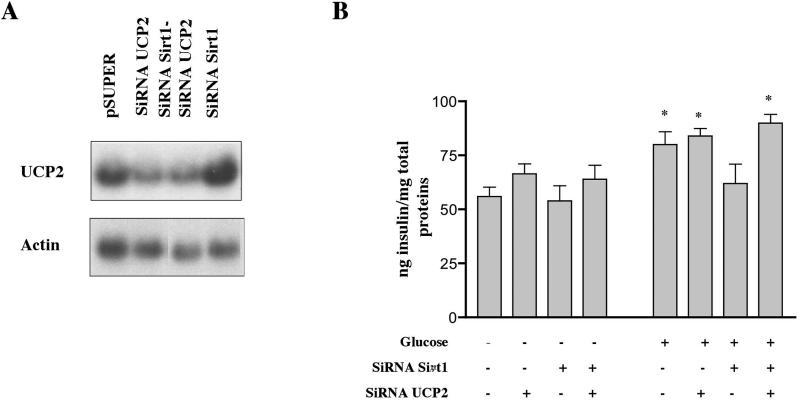
Knockdown of UCP2 in Sirt1 Knockdown Cells Restores Glucose-Induced Insulin Secretion (A) Northern blot for UCP2 RNA in control INS-1 cells, and cells knocked down for Sirt1 (SiRNA Sirt1), UCP2 (SiRNA UCP2), or both Sirt1 and UCP2 (SiRNA Sirt1-SiRNA UCP2). RNAs were quantitated by densitometry, setting the level of UCP2 in control cells at 1.0. (B) Insulin secretion in INS-1 control cells and cells with knockdown levels of Sirt1, UCP2, or both Sirt1 and UCP2 after treatment with 16.7 mM glucose (+) or 4mM glucose (−) for 1 h (*n* = 3 experiments done in triplicate, *p < 0.05 in SiRNA Sirt1-SiRNA UCP2, ANOVA).

The above findings show that Sirt1 represses UCP2, and alleviation of this repression correlated with blunted insulin secretion in response to glucose. To address whether the increase in UCP2 in Sirt1 knockdown cells *caused* the failure to secrete insulin, UCP2 was also knocked down in INS-1 cells with reduced Sirt1. Stable cell lines with UCP2 knocked down by SiRNA were derived from two different lines in which Sirt1 was already knocked down, as well as from control INS-1 cells. Several different UCP2 sequences were inserted into a pSUPER hairpin vector with the neomycin-resistance drug marker. These constructs were transfected into the SiRNA Sirt1 puromycin resistant cells or control cells, and stable populations of NeoR cells were assayed for UCP2 RNA and protein by Northern and Western blots. In the case of two different UCP2 SiRNA constructs, the levels of UCP2 RNA (Figure 6A) and protein (unpublished data) were clearly reduced in transfected cells. The levels of Sirt1 remained low in the double knockdown cells (unpublished data).

Next, glucose-stimulated insulin secretion was assayed in Sirt1 or UCP2 knockdown cells and in cells with both Sirt1 and UCP2 knocked down. The Sirt1 knockdown cells were again defective in induction of insulin secretion, as expected. However, the double knockdown cells or cells with only the UCP2 SiRNA construct displayed insulin secretion in response to glucose (Figure 6B). A similar effect was observed in the second Sirt1 knockdown line in which UCP2 was also knocked down (unpublished data). This result demonstrates that the failure of Sirt1 knockdown cells to secrete insulin is due to the elevated levels of UCP2 in these cells. We conclude that Sirt1 acts as a positive regulator of insulin secretion in wild-type cells by repressing UCP2, thereby allowing coupling of glucose metabolism to ATP synthesis.

### UCP2 Levels Increase in Food-Deprived Mice

**Figure 7:**
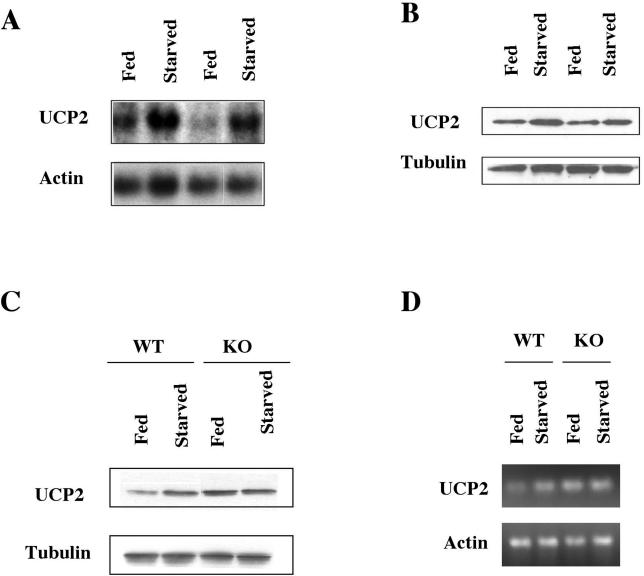
UCP2 mRNA or Protein Levels in Fed or Starved Wild-Type Mice (A) Northern blot for UCP2 in whole pancreas of two *ad libitum* mice and two mice starved for 18 h. (B) Western blot for UCP2 in isolated islets in two *ad libitum* and two starved mice. (C) Western blot for UCP2 in wild-type (WT) or Sirt1 KO littermates either fed *ad libitum* or starved for 18 h. The experiment shown is representative of four pairs of wild-type and KO littermates analyzed. (D) RT-PCR for UCP2 in wild-type or Sirt1 KO mice fed or starved.

Does the regulation of UCP2 play any role in the normal secretion of insulin in β cells of wild-type mice in response to diet? To address this question, we starved wild-type mice O/N and compared the levels of UCP2 in whole pancreas and in islets to mice feeding *ad libitum.* Importantly, we found an increase in UCP2 mRNA levels in whole pancreas of starved mice compared with the fed mice (Figure 7A). Further, the levels of UCP2 protein were also increased in starved mice (Figure 7B).

In order to determine whether Sirt1 regulated this induction of UCP2 in mice, we starved Sirt1 KO mice O/N and compared the effect of starvation on UCP2 protein levels to wild-type mice. Four pairs of wild-type and KO littermates were compared and gave comparable results. The levels of UCP2 in KO mice fed *ad libitum* were elevated compared with wild-type, as expected (Figure 7C). Most importantly, these elevated levels in the fed KO mice were not further induced by starvation. In contrast, starvation induced UCP2 in wild-type mice, as before. A similar pattern of UCP2 RNA induction by starvation in wild-type but not KO mice was observed by RT-PCR (Figure 7D). The above findings suggest that an increase in UCP2 in β cells is part of a normal mechanism to regulate the capacity of β cells to produce insulin. Moreover, this induction appears to be mediated by alleviation of Sirt1-mediated repression.

**Figure 8:**
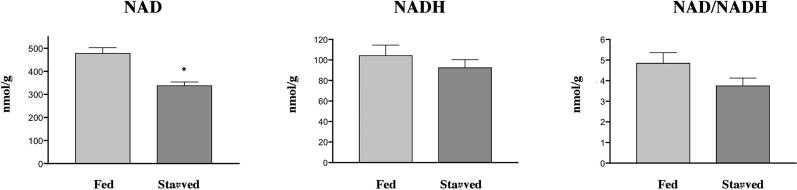
NAD and NADH Levels in Fed and Starved Mice Measurements were made in pancreases of seven fed and seven starved wild-type mice, and levels are expressed as nmol per gram of tissue. The decrease in NAD in starved mice is significant with *p* < 0.0005, while the NADH levels in fed versus starved are not significantly different.

These findings suggest that starvation causes a decrease in Sirt1 activity in β cells. In yeast, Sir2p activity is regulated by the NAD/NADH ratio. We thus measured NAD and NADH in the pancreas of seven fed and seven starved wild-type mice. Strikingly, there was a significant decrease in the level of NAD but not NADH in the starved mice (Figure 8). The level of Sirt1 protein in starved pancreas is roughly comparable to fed controls (unpublished data). These findings suggest that changes in NAD levels in the pancreas regulate Sirt1 activity and insulin secretion in response to diet.

## Discussion

In this study we show that Sirt1 regulates insulin secretion in pancreatic β cells. Both in Sirt1 KO mice and in cultured β cell lines, a reduction in Sirt1 levels reduces the capacity of these cells to secrete insulin in response to glucose. Classical studies show that an increase in the ATP/ADP ratio due to glucose metabolism is the critical trigger in the induction of insulin secretion. In cells with reduced Sirt1, the increase in this ratio is blunted due to elevated levels of UCP2, which reduces the synthesis of ATP during respiration. Sirt1 directly represses UCP2 by binding to the promoter. Thus, by controlling UCP2, Sirt1 regulates the amplitude of insulin induction by glucose. We do not know whether the low levels of insulin in the whole body of Sirt1 KO mice represents a β cell defect, an indirect consequence of the elevated glucose tolerance in these animals, or both.

The use of UCP2 to regulate insulin production is distinct from the canonical role of UCPs. By uncoupling respiration from ATP synthesis in brown fat, for example, UCP1 generates heat and is critical for the non-shivering thermogenesis in rodents (reviewed in [48]). Here UCP2 is used to modulate the levels of ATP made in β cells, which dictates the extent of insulin secretion. Our findings are consistent with the elevated basal levels of insulin observed in fasted UCP2 KO mice [36] and suggest that Sirt1 and UCP2 are physiologically relevant regulators of insulin production (see “UCPs and Longevity–Cellular Basis”).

### Physiology of Insulin Regulation by Sirt1

The fact that Sirt1 represses UCP2 expression in β cells is intriguing in light of the role of this sirtuin as a regulator of CR in lower organisms. Our findings indicate that Sirt1 may link the amplitude of insulin secretion to the diet. We found that O/N starvation led to an increase in the levels of UCP2 protein and RNA in the pancreas and isolated islets. This increase did not occur in Sirt1 KO mice, which already displayed higher levels of UCP2 when fed *ad libitum*. These findings suggest that regulation of UCP2 by Sirt1 is a physiologically important mechanism to blunt the chronic levels of insulin in starved animals. This mechanism may explain why insulin levels are so low during fasting, even though β cells are actively metabolizing fat and respiring (see following section). It remains to be seen whether this mechanism is important during long-term CR.

### Regulation of Pancreatic Sirt1 Activity by NAD/NADH and Diet

The action of mammalian Sirt1 appears to differ from that of lower organisms. In *C. elegans, sir2.1* appears to repress the output of the insulin/IGF pathway [9], but the mechanism has not been described. Also, Sir2 activity is activated during CR to extend the life span in yeast and Drosophila [11,49]. However, in mammals Sirt1 functions as a positive regulator of insulin secretion, raising the possibility that its activity in β cells is actually *reduced* by O/N starvation..

A lowering of Sirt1 activity occurs concomitant with the shift from carbohydrate-based metabolism to utilization of fatty acids, which is known to follow food deprivation. Because fatty acids are more reduced than carbohydrates, their metabolism to CO_2_ converts more NAD to NADH. Indeed, we show that the NAD/NADH ratio falls in pancreas of starved mice compared with fed controls, although we cannot be certain this change occurs specifically in islets, which represent a small percentage of pancreatic mass. A decrease in the NAD/NADH ratio has also been shown to down-regulate Sir2 activity in other physiological contexts [15,21,50].

### UCPs and Longevity–Cellular Basis

The question arises whether the up-regulation of UCPs we find in β cells occurs in other tissues during food limitation or long-term CR. In a horizontal study of mice, a good correlation was found between the life span and the level of uncoupling of respiration [51]. It has also been shown that overexpression of a UCP increased the life span in Drosophila [52]. UCPs may confer benefit by damping production of reactive oxygen species during respiration. In one example, mitochondria from UCP3 KO mice generated higher levels of superoxide [53,54], suggesting that some degree of uncoupling can be beneficial by controlling reactive oxygen species production [55].

There are several reports that levels of UCPs may increase as a consequence of CR [56,57], but these findings are still not conclusive [58]. It will be interesting to see whether Sirt1 regulates UCPs in tissues other than β cells, and whether there is a broader change in the expression of UCPs during acute food limitation or CR.

### Summary and Perspective

The induction of insulin secretion by glucose in pancreatic β cells has been a paradigm in mammalian cellular physiology. Here we describe a new level of regulation in which Sirt1 represses UCP2 to modulate the amplitude of insulin induction by glucose in β cells. We suggest that this mechanism serves to regulate chronic levels of insulin in accord with levels of food intake. This mechanism may reinforce the low levels of insulin production during food limitation and coordinate insulin release from β cells to the insulin sensitivity of metabolic tissues set by the diet. Regulation of UCP2 by Sirt1 may also be an important axis that is dysregulated by excess fat to contribute to obesity-induced diabetes.

## Materials and Methods

### Cell culture

INS-1 (a gift from K. Olson, Michigan State University, Lansing, Michigan, United States) and MIN6 cells (a gift from S. Imai, Washington University in St. Louis, St. Louis, Missouri, United States) were grown at 5% CO_2_/95% air at 37 °C. INS-1 cells were cultured in RPMI-1640 medium containing 11.1 mM glucose supplemented with 10% fetal bovine serum (FBS), 1 mM pyruvate, 10 mM HEPES, 50 μM 2-mercaptoethanol, 100 U penicillin/ml, and 100 μg/ml streptomycin. Cells were passaged weekly after trypsin-EDTA treatment. All studies were performed in INS-1 passages between 70 and 84. MIN6 cells were grown in Dulbecco's modified Eagle's medium (DMEM) containing 4.5 g/l glucose and L-glutamine, 15% FBS, 3.4 g/l sodium bicarbonate, 5 μl/l 2-mercaptoethanol, 100 U penicillin/ml, and 100 μg/ml streptomycin. All studies were performed between passages 27 and 35.

### Animal experimentation

The Sirt1 KO animals and their controls were obtained from M. McBurney [39]. The mice were housed under controlled conditions: temperature (25 ± 1 °C) and light cycle (7 a.m.–7 p.m.). Animals were fed with regular chow and cared for in accordance with the MIT Committee on Animal Care (Massachusetts Institute of Technology, Cambridge, Massachusetts, United States). Assays were performed on 4- to 8-mo-old males (129/Sv CD1 mixed background).

### Retroviral infection of INS-1 and MIN6 cells

Phoenix cells were transfected with either pBABE, pBABE-T1 [18], pSUPERretro (Oligogene), pSUPERretro-SiRNA-T1 (5′- GCTGCATCCAAGGGCCATG-3′), or pSUPER retro SiRNA-GFP (gift from T. Brummelkamp, Whitehead Institute for Biomedical Research, Cambridge, United States of America) using Lipofectamine 2000 (Invitrogen, Carlsbad, California, United States). 48 h post-transfection, the medium containing retroviruses was collected and added with 1 mg/ml polybrene to INS-1 or MIN6 cells. Infected cells were treated with 1 mg/ml of puromycin for selection.

In order to create double knockdown cell lines, INS-1 cells stably transfected with control vector and pSUPERretro-SiRNA-T1 were infected with retroviruses carrying pSUPER retro-SiRNA UCP2. These cells were selected with 200 μg/ml gentamicin (Mediatech, Herndon, Virginia, United States).

### Cell-growth analysis

INS-1 cells were seeded at 1 × 10^5^ cells/6 cm plate. Cells were collected, resuspended in the same volume of medium, and counted using the trypan blue exclusion method.

### Insulin secretion assay

0.5 × 10^6^ cells were placed in 12-well plates in growing medium. In the case of INS-1 cells, fresh medium containing 4.0 mM glucose was added after 2 d. Cells were cultured for an additional day. In all cases, the medium was removed on the day of the experiment, and the cells washed three times with warm KRB buffer (119 mM NaCl, 4.74 mM KCl, 2.54 mM CaCl_2_, 1.19 mM MgS0_4_, 1.19 mM KH_2_P0_4_, 25 mM NaHC0_3_, 10 mM HEPES [pH 7.4], 0.1 g BSA). The cells were then incubated with 1 ml of KRB at 37 °C for 60 min. At the end of the incubation, cells were washed with KRB medium. The cells were then incubated with KRB containing different glucose concentrations (4–16.7 mM for INS-1 and 4–20 mM for MIN6) for 1 h at 37 °C. The supernatant was collected and insulin was measured by ELISA using mouse insulin as a standard (Alpco Diagnostics, Windham, New Hampshire, United States). Cells were then incubated O/N with acidified EtOH (75% EtOH, 1.5% HCl). Protein determination was performed using the Bio-Rad Dc Protein assay (Hercules, California, United States). The amounts of secreted insulin were corrected by the amounts of cell protein in each well.

### Immunofluorescence

Pancreas was isolated from wild-type or Sirt1 KO mice and fixed in 4% paraformaldehyde. Consecutive 8-μm sections were immunostained with anti-Sirt1 antibody at 1:100 dilution (Upstate-Chemicon, Charlottesville, Virginia, United States). DAPI was used to stain the nuclei. Hematoxylin and eosin was used to visualize islets.

### In vivo insulin measurement

Mice were subjected to O/N fast followed by intraperitoneal glucose injection (1 g/kg body weight). Blood samples were collected from the tail vein the night before the experiment *(ad libitum),* right before the glucose injection (time 0), and after the different time points indicated in Figure 2E. Insulin levels were measured using the Ultrasensitive Mouse Insulin EIA (Alpco Diagnostics) according to manufacturer's specifications.

### Glucose tolerance test

Mice were fasted O/N (16 h) and injected intraperitoneally with a saline glucose solution at 1g/kg body weight. Plasma glucose levels were measured from tail blood before and 2, 5, 10, 25, 40, 80, and 120 min after the glucose injection. The test was repeated five times with results comparable to those in Figure 2D.

### Western blot analysis

Cells were lysed in RIPA buffer (1% TritonX-100, 158 mM NaCl, 5 mM EDTA, 10 mM Tris [pH 7.0], protease inhibitors [Boehringer, Mannheim, Germany], 1 mM DTT, and 0.1% phenylmethylsulfonyl fluoride), sonicated, and centrifuged at 14,000 rpm for 10 min. Proteins were separated by SDS-PAGE, transferred to nitrocellulose membrane (Schleicher and Schuell Bioscience, Dassel, Germany), and were probed with a polyclonal antibody against Sirt1 or monoclonal anti-actin antibody (Chemicon, Temecula, California, United States). The UCP2 antibody (Calbiochem, San Diego, California, United States) was used in 1:1000 dilution. The cEBP/β, the insulin receptor alpha and beta, and the kir6.2 antibodies (Santa Cruz Biotechnology, Santa Cruz, California, United States) were used at 1:200, 1:200, 1:500, and 1:200, respectively.

### Chromatin immunoprecipitation

Assays were performed on cells as previously described according to the Farnham protocol (http://mcardle.oncology.wisc.edu). Immunoprecipitations were performed with 1μg of Sirt1 antibody. PCR primers were designed to span a regulatory region in the UCP2 promoter (5′- CGTCTGTTCAAAGCGTCTCA; 5′- CCAGCTGGAGTCTTCTCCTT) (set 5) or a control region in the UCP2 promoter (5′- AGGTTGTTTCTGGGCCATGTGCTCTAA; 5′- TAGACCCTGGCCACCCTGAGCGCGAAAT) (set 4).

### Islets isolation and morphometric analysis

Islets were isolated using a collagenase technique as described previously [59]. The pancreas was dissected and islets separated by incubation with collagenase at 37 °C. Islets were then hand-picked using a microscope. Islets were used for Western blot analyses or for glucose-induced insulin secretion assays. To measure insulin secretion, islets were incubated in KRB with 4 mM glucose for 1 h and then incubated with KRB containing different glucose concentrations (4–20 mM) for 1 h at 37 °C. The experiment was carried out twice with four mice per treatment, and the repeat experiment gave data comparable to Figure 2E.

Four to five pancreases each from WT, Sir2+/−, and Sir2−/− mice were fixed in 2% chilled paraformaldehyde, and paraffin embedded, and three sections (5 μm) separated by at least 100 μm were dewaxed using xylene, rehydrated through serial dilutions of ethyl-alcohol, and subjected to antigen retrieval using 10 mM citrate (pH 6.1), followed by DAKO high pH antigen retrieval solution (Dako, Glostrup, Denmark). The sections were washed and stained with the respective antibodies in staining buffer with 100 mM NaCl, 3% BSA, 0.5% Triton-X-100, and 50 mM Na-PO_4_ (pH 7.4). Primary antibodies included sheep anti-insulin (The Binding Site, Birmingham, United Kingdom), mouse anti-glucagon (Sigma, St. Louis, Missouri, United States), and rabbit anti-somatostatin (Dako). The entire pancreatic section of each stained slide was arbitrarily divided into non-overlapping contiguous portions (using a microscope inset measuring guide) and imaged at 10× magnification. The islet area (in square micrometers) and the total area of each section were determined by tracing the outline to assess the area of the islets, using image analyzer software (Image-Pro 4.1 Plus). The percentage of islet cell area in the pancreas was expressed as a percentage of the total pancreatic area. For 4× images, islets were stained using a cocktail of antibodies against insulin, glucagon, and somatostatin and HRP-linked secondary antibodies with DAB as a chromogen.

### ATP/ADP measurements

1 × 10^6^ cells were washed with 1× PBS. Ice-cold 6% (v/v) HClO_4_ was added, and immediately the cells were scraped from the plate. The solution was neutralized with 1 M KHCO_3_, centrifuged briefly, and the supernatant was passed through a 0.2-m filter (Nanosep, Lund, Sweden), and subjected to reversed phase chromatography using a Targa C18 250 × 4.6 mm 5-mm column as described in [61]. Nucleotides were detected at 260 nm with a Waters 486 tunable detector. Peak heights were measured. Nucleotide identities were confirmed by co-migration with known standards.

### RNA analyses

Northern blot—Total RNA from INS-1 cells was extracted using Triazol reagent (Invitrogen). For Northern blot analysis, 10 μg of RNA samples was separated on a formaldehyde gel, transferred to nylon membrane, and then hybridized with gene-specific probes. Normalization of mRNA was done by using actin as probe (kind gift from K. Olson). For whole-pancreas RNA extraction, fresh pancreas was stored in the RNA later solution (Ambion, Austin, Texas, United States) before extracting total RNA with the Qiagen system (Qiagen, Valencia, California, United States). The UCP2 probe was a kind gift from D. Ricquier, CNRS, Paris, France. RT-PCR/cDNA was synthesized from 1 μg of total RNA from the pancreas. The reverse transcriptase reaction was performed at 25 °C for 10 min, 42 °C for 1 h, 95 °C for 5 min, for one cycle. The PCR reaction (95 °C for 30 s, 58 °C for 30 s, 72 °C for 30 s, for 25 cycles) was performed using the respective sense and antisense oligonucleotide primers: UCP2: 5′- GCCCGGGCTGGTGGTGGTC and 5′- CCCCGAAGGCAGAAGTGAAGTGG; β-actin: 5′- GAACCCTAAGGCCAACCGTGAAAAGAT and 5′- ACCGCTCGTTGCCAATAGTGATG.

### CAT assay

Transfections for CAT assays were done as described [60]. 293T cells were transiently transfected with pSU2N2-mUCP2-CAT (which contains 7 kb of the mouse UCP2 promoter; gift from D. Ricquier), pCMV-betaGal (to correct for transfection efficiency), and either pCMV-mSirt1 and pSPORT6-mPPAR-gamma2 (gift from B. Spiegelman, Harvard Medical School, Massachusetts, United States of America) or their respective empty vectors. 24 h later, total CAT enzyme levels were measured by ELISA (Roche, Basel, Switzerland).

### NAD and NADH determination

NAD and NADH nucleotides were measured as described [15]. About 10 mg of frozen pancreas tissues was homogenized in 300 μl of acid extraction buffer to obtain NAD concentration, or alkali buffer to obtain NADH concentration. 240 μl of supernatant was neutralized with 120 μl of buffer. The concentration of nucleotides was measured fluorimetrically after an enzymatic cycling reaction using 2 μl of sample. All values were detected within the linear range and are expressed as nmol per gram of tissue.

### Quantitation of NADH autofluorescence and glucose uptake

1.5 × 10^6^ INS-1 cells were seeded in six-well plates. Fresh medium containing 4.0 mM glucose was added the day before the experiment. The medium was removed on the day of the experiment, and the cells washed 3× with warm KRB buffer. The cells were then incubated with 1 ml of KRB at 37 °C for 1 h. At the end of the incubation, the media was removed and the cells were incubated for 10 min with KRB containing different glucose concentrations: 4–16.7 mM (for NADH determination), or 200 μM 2-NBDG (Molecular Probes) for glucose uptake determination. At the end of the incubation, cells were washed 3× with cold 1× PBS. We then used a four-laser LSR II digital flow cytometer (Becton-Dickinson, Mountain View, California, United States) with Diva software. The 488-mm laser excited the fluorescent glucose, and emission was detected with a 530/30BP filter and expressed as arbitrary units. NADH was measured according to Thorell [46] with a 355-mm laser and detected with a 440/40BP filter. Cell viability was assessed using PI. Data were analyzed with FlowJo (Tree Star, Ashland, Oregon, United States).

## Supporting Information

Figure S1 Growth Curve Plot of Cell Number vs. Time of INS-1 Cells Transfected with pSUPER (black line) or SiRNA Sirt1 (red line) (163 KB TIF).

## Abbreviations

CAT: chloramphenicol acetyl-transferase
CR: calorie restriction
KO: knockout
NAD: a derivative of niacin
NADH: the reduced form of NAD
O/N: overnight
*Sir2*: silent mating type information regulation 2
Sirt1: Homolog of the yeast silencing information regulator2
UCP: uncoupling protein
WAT: white adipose tissue
