# Correction: Sirt1 Regulates Insulin Secretion by Repressing UCP2 in Pancreatic ß Cells

**DOI:** 10.1371/journal.pbio.0040295

**Published:** 2006-09-12

**Authors:** Laura Bordone, Maria Carla Motta, Frederic Picard, Ashley Robinson, Ulupi S Jhala, Javier Apfeld, Thomas McDonagh, Madeleine Lemieux, Michael McBurney, Akos Szilvasi, Erin J Easlon, Su-Ju Lin, Leonard Guarente

In *PLoS Biology*, volume 4, issue 2: DOI: 10.1371/journal.pbio.0040031

In the first paragraph of the Materials and Methods subsection “Retroviral infection of INS1 and MIN6 cells,” “pSUPERretro SiRNA-T1 (5′-GCTGCATCCAAGGGCCATG-3′)” should be “pSUPERretro SiRNA-T1 (5′-gatgaagttgacctcctca-3′)”.

