# Correction: Sirt1 Regulates Insulin Secretion by Repressing UCP2 in Pancreatic β Cells

**DOI:** 10.1371/journal.pbio.1002346

**Published:** 2015-12-29

**Authors:** Laura Bordone, Maria Carla Motta, Frederic Picard, Ashley Robinson, Ulupi S. Jhala, Javier Apfeld, Thomas McDonagh, Madeleine Lemieux, Michael McBurney, Akos Szilvasi, Erin J. Easlon, Su-Ju Lin, Leonard Guarente

The authors would like to clarify that the controls previously depicted in Figs 4E and 7A were for different experiments and were included in error.

**Fig 4:**
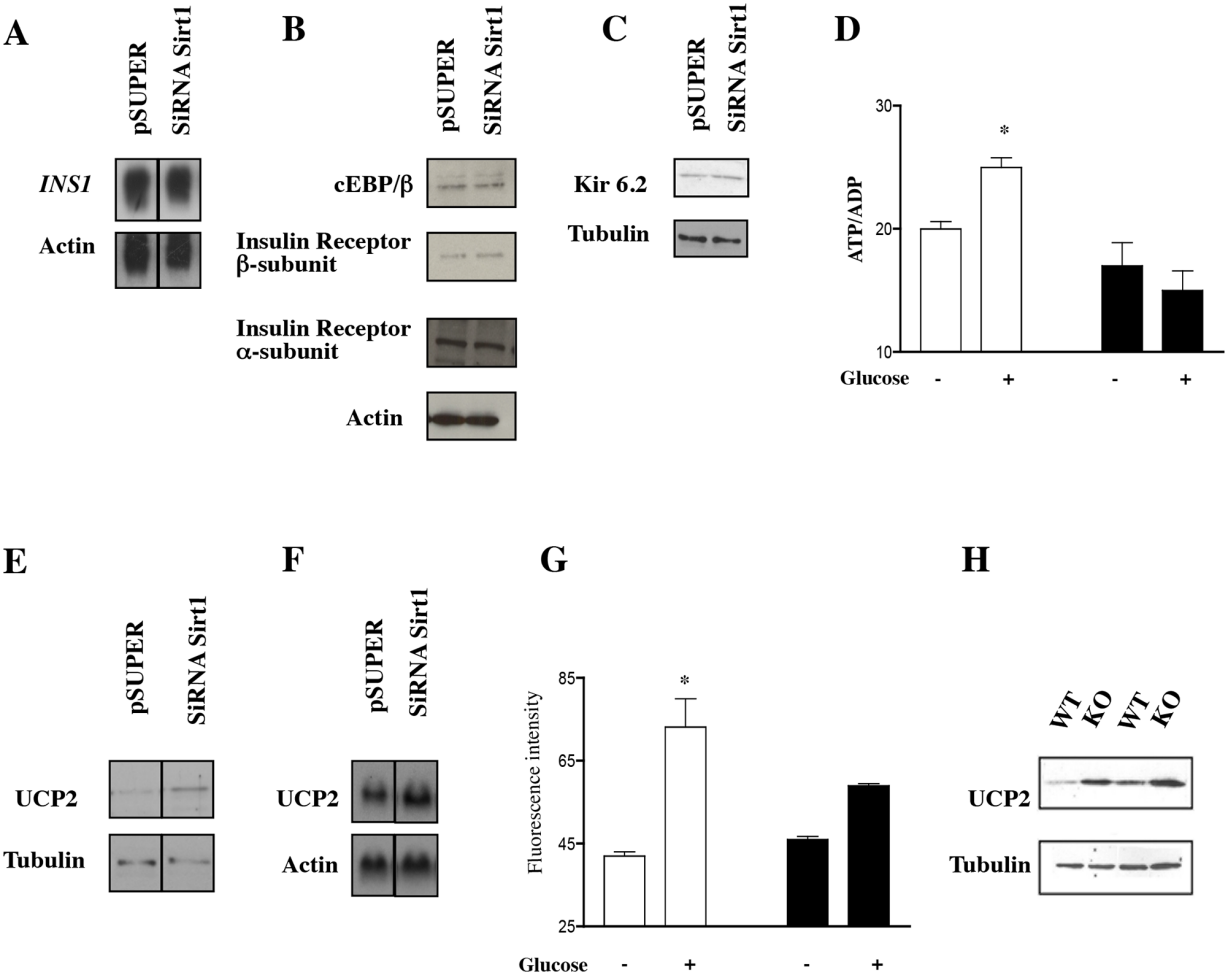
UCP2 is Up-Regulated in Sirt1 Knockdown Cells and in Sirt1 KO Mice. (A) Northern blot for the insulin gene INS-1 in cells with a control vector (pSUPER) or a SiRNA-Sirt1 vector (SiRNA Sirt1). Vertical lines indicate where the respective original blots were spliced together. (B and C) Western blot analyses of targets involved in insulin synthesis and secretion using specific antibodies: cEBP/β, insulin receptor α and β, and kir6.2, one of the K^+^channel receptor subunits. (D) Measurement of ATP/ADP levels in INS-1 control cells (open bars) or Sirt1 knockdown cells (black bars) treated with 16.7 mM glucose (+) or 4 mM glucose (−) (*n* = 3 experiments done in triplicate, *p < 0.005 in the pSUPER experiment; ANOVA). (E) Western blot analysis for UCP2 in INS-1 control cells (pSUPER) or knockdown cells (SiRNA Sirt1). Vertical lines indicate where the respective original blots were spliced together. (F) Northern blot analysis for UCP2 in INS-1 control cells (pSUPER) or knockdown cells (SiRNA Sirt1). Vertical lines indicate where the respective original blots were spliced together. (G) NADH levels in INS-1 cells after glucose addition as determined by autofluorescence [46] and expressed as arbitrary units. Cells stably transfected with control or Sirt1 SiRNA vectors were used (*n* = 2, *p < 0.05 compared with no glucose). (H) UCP2 protein levels in isolated pancreatic islets of two wild-type or two Sirt1 KO mice. Tubulin or actin was used as loading control in all Western and Northern blots.

The correct control for Fig 4E was located and used to prepare a corrected figure.

The correct control for the original Fig 7A could not be located; this panel has therefore been removed after a careful assessment and investigation determined that the result for which original Fig 7A was cited is supported elsewhere in this article, and that removal of this panel does not affect the conclusions of the paper.

We have also taken this opportunity to provide new versions of several figures (Figs 4, 5, 6, 7) in which gel/blot splices and a non-linear level adjustment were made but were not previously indicated or declared, or to replace incorrectly spliced gels/blots with the un-spliced originals. We also take the opportunity to correct two errors in the legend to Fig 6, first to remove a redundant and incorrect sentence, and second to address incorrect description of *p* values.

**Fig 5:**
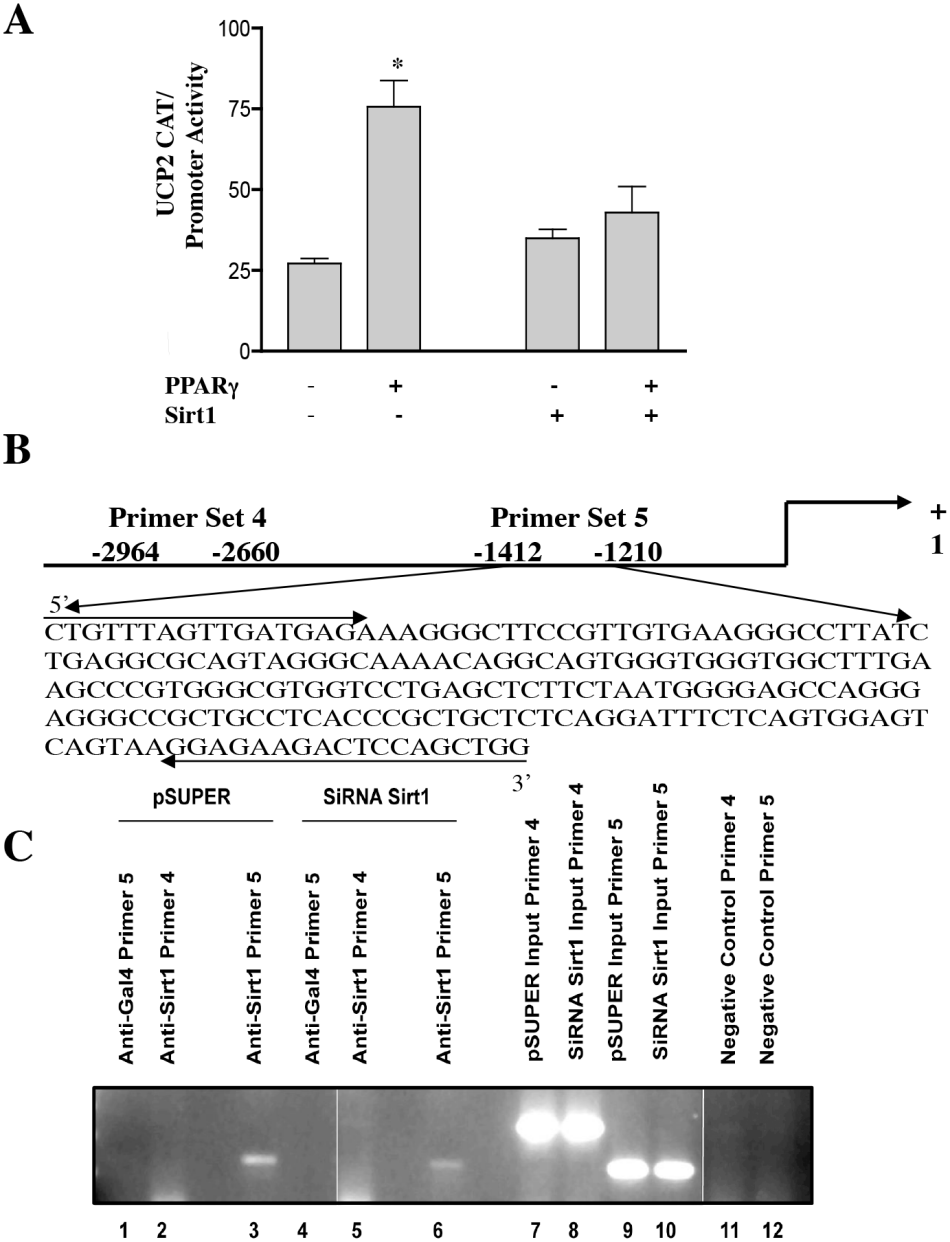
Sirt1 Binds at the UCP2 Promoter and Represses the Gene. (A) In vitro CAT assay. 293T cells were transfected with a CAT reporter driven by the UCP2 promoter. Cells were also co-transfected with Sirt1 or not and with PPARγ or not, as indicated. CAT activity was determined (*n* = 3 experiments done in triplicate, *p < 0.05 in the no Sirt1 transfection experiment, ANOVA). (B) Schematic representation of the primer sets (arrows) in the UCP2 promoter (shown schematically and with excerpted DNA sequence). (C) Chromatin-immunoprecipitation (IP) was carried out on INS-1 control cells (lanes 1–3) or Sirt1 knockdown cells (columns 4–6) using Sirt1 antibody or a Gal4 control antibody, as indicated. PCR was carried out with the indicated primers. INPUT (columns 7–10) refers to PCR carried out on samples prepared prior to immunoprecipitation. Negative controls for the PCR (minus DNA) are also indicated (columns 11 and 12). In preparing the original panel for publication, the shadow/midtone/highlight input levels in the gray channel were adjusted uniformly to approximately 35/1.00/85 units. Vertical lines indicate where the original gel image was spliced together.

**Fig 6:**
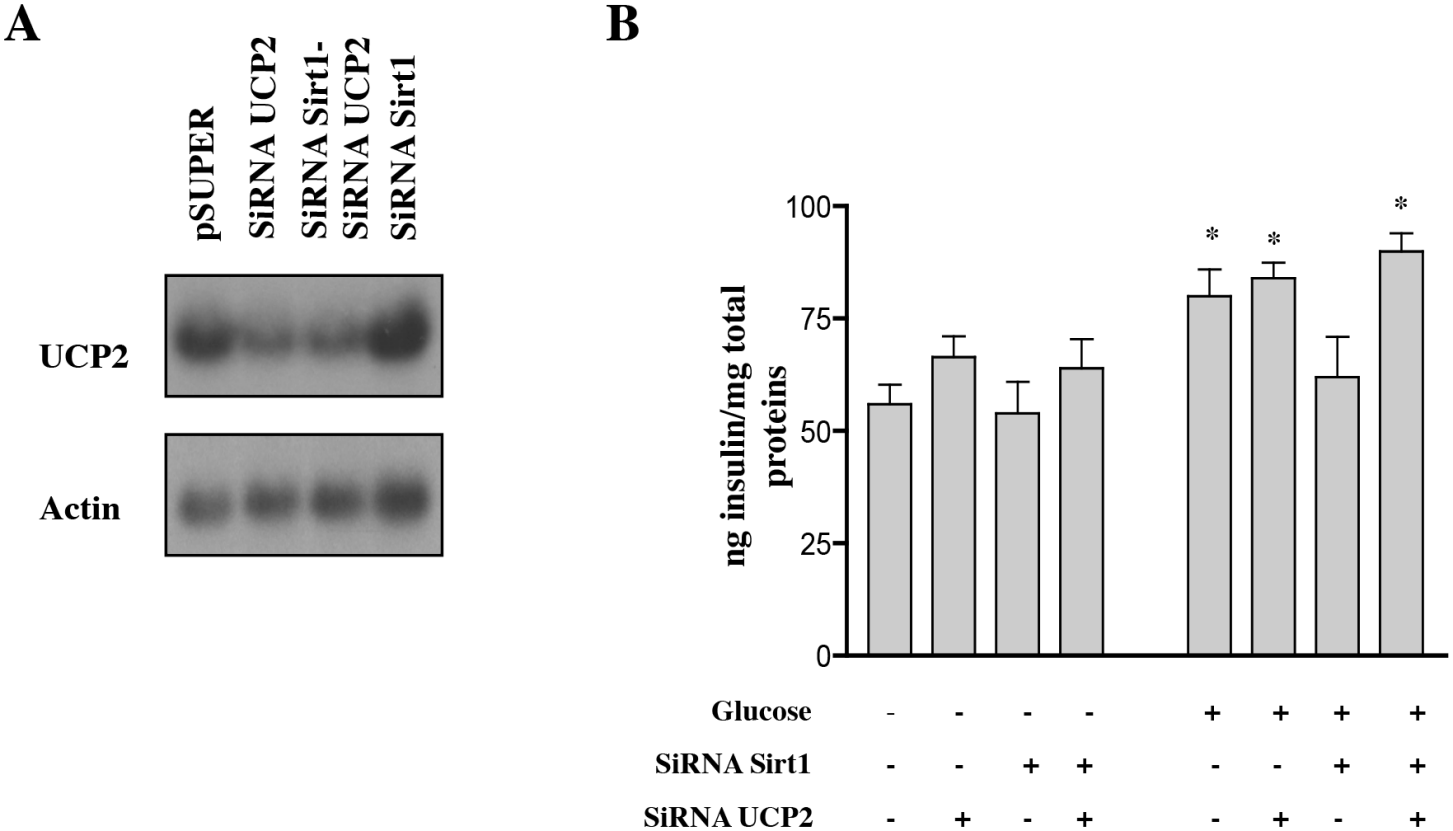
Knockdown of UCP2 in Sirt1 Knockdown Cells Restores Glucose-Induced Insulin Secretion. (A) Northern blot for UCP2 RNA in control INS-1 cells, and cells knocked down for Sirt1 (SiRNA Sirt1), UCP2 (SiRNA UCP2), or both Sirt1 and UCP2 (SiRNA Sirt1-SiRNA UCP2). (B) Insulin secretion in INS-1 control cells and cells with knockdown levels of Sirt1, UCP2, or both Sirt1 and UCP2 after treatment with 16.7 mM glucose (+) or 4mM glucose (−) for 1 h (*n* = 3 experiments done in triplicate, *p < 0.05 compared to no glucose, ANOVA).

**Fig 7:**
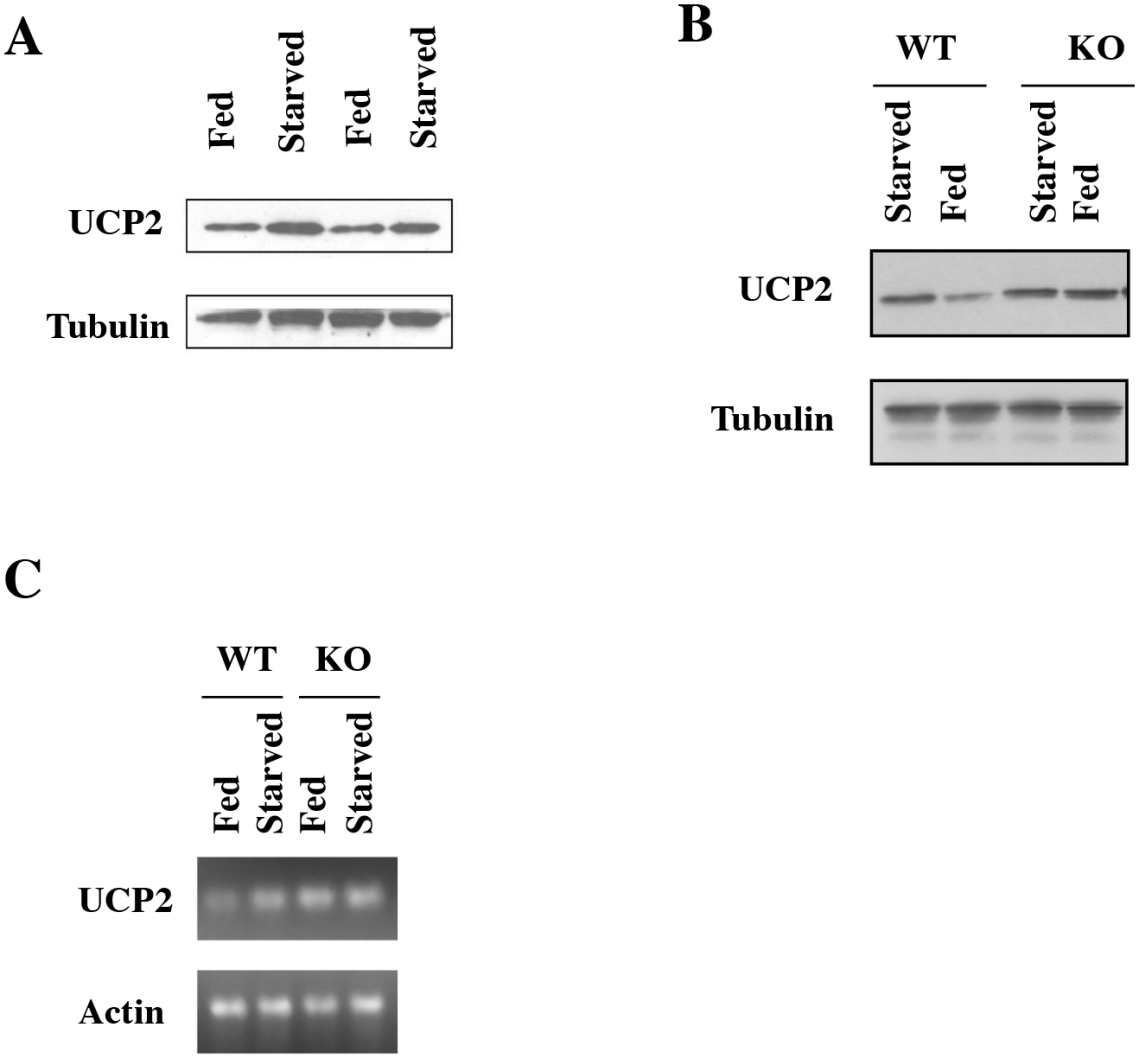
UCP2 mRNA or Protein Levels in Fed or Starved Wild-Type Mice. Western blot for UCP2 in isolated islets in two *ad libitum* and two starved mice. (B) Western blot for UCP2 in wild-type (WT) or Sirt1 KO littermates either fed *ad libitum* or starved for 18 h. The experiment shown is representative of four pairs of wild-type and KO littermates analyzed. (C) RT-PCR for UCP2 in wild-type or Sirt1 KO mice fed or starved.

The text in the Results section titled “UCP2 Levels Increase in Food-Deprived Mice” has been edited to accommodate the removal of the original Fig 7A and the relabeling of Fig 7B, 7C and 7D as Fig 7A, 7B and 7C, respectively. The corrected text and Figs 4, 5, 6 and 7 are provided here.

## UCP2 Levels Increase in Food-Deprived Mice

Does the regulation of UCP2 play any role in the normal secretion of insulin in β cells of wild-type mice in response to diet? To address this question, we starved wild-type mice O/N and compared the levels of UCP2 in whole pancreas and in islets to mice feeding *ad libitum*. Importantly, we found an increase in levels of UCP2 protein in starved mice compared with fed mice (Fig 7A).

In order to determine whether Sirt1 regulated this induction of UCP2 in mice, we starved Sirt1 KO mice O/N and compared the effect of starvation on UCP2 protein levels to wild-type mice. Four pairs of wild-type and KO littermates were compared and gave comparable results. The levels of UCP2 in KO mice fed *ad libitum* were elevated compared with wild-type, as expected (Fig 7B). Most importantly, these elevated levels in the fed KO mice were not further induced by starvation. In contrast, starvation induced UCP2 in wild-type mice, as before. A similar pattern of UCP2 RNA induction by starvation in wild-type but not KO mice was observed by RT-PCR (Fig 7C). The above findings suggest that an increase in UCP2 in β cells is part of a normal mechanism to regulate the capacity of β cells to produce insulin. Moreover, this induction appears to be mediated by alleviation of Sirt1-mediated repression.

These findings suggest that starvation causes a decrease in Sirt1 activity in β cells. In yeast, Sir2p activity is regulated by the NAD/NADH ratio. We thus measured NAD and NADH in the pancreas of seven fed and seven starved wild-type mice. Strikingly, there was a significant decrease in the level of NAD but not NADH in the starved mice (Fig 8). The level of Sirt1 protein in starved pancreas is roughly comparable to fed controls (unpublished data). These findings suggest that changes in NAD levels in the pancreas regulate Sirt1 activity and insulin secretion in response to diet.
